# Markup: A Web-Based Annotation Tool Powered by Active Learning

**DOI:** 10.3389/fdgth.2021.598916

**Published:** 2021-07-26

**Authors:** Samuel Dobbie, Huw Strafford, W. Owen Pickrell, Beata Fonferko-Shadrach, Carys Jones, Ashley Akbari, Simon Thompson, Arron Lacey

**Affiliations:** ^1^Health Data Research UK, Swansea University Medical School, Swansea University, Swansea, United Kingdom; ^2^Swansea University Medical School, Swansea University, Swansea, United Kingdom; ^3^Neurology Department, Morriston Hospital, Swansea Bay University Health Board, Swansea, United Kingdom

**Keywords:** natural language processing, active learning, unstructured text, annotation, sequence-to-sequence learning

## Abstract

Across various domains, such as health and social care, law, news, and social media, there are increasing quantities of unstructured texts being produced. These potential data sources often contain rich information that could be used for domain-specific and research purposes. However, the unstructured nature of free-text data poses a significant challenge for its utilisation due to the necessity of substantial manual intervention from domain-experts to label embedded information. Annotation tools can assist with this process by providing functionality that enables the accurate capture and transformation of unstructured texts into structured annotations, which can be used individually, or as part of larger Natural Language Processing (NLP) pipelines. We present Markup (https://www.getmarkup.com/) an open-source, web-based annotation tool that is undergoing continued development for use across all domains. Markup incorporates NLP and Active Learning (AL) technologies to enable rapid and accurate annotation using custom user configurations, predictive annotation suggestions, and automated mapping suggestions to both domain-specific ontologies, such as the Unified Medical Language System (UMLS), and custom, user-defined ontologies. We demonstrate a real-world use case of how Markup has been used in a healthcare setting to annotate structured information from unstructured clinic letters, where captured annotations were used to build and test NLP applications.

## Introduction

**Figure 1:**
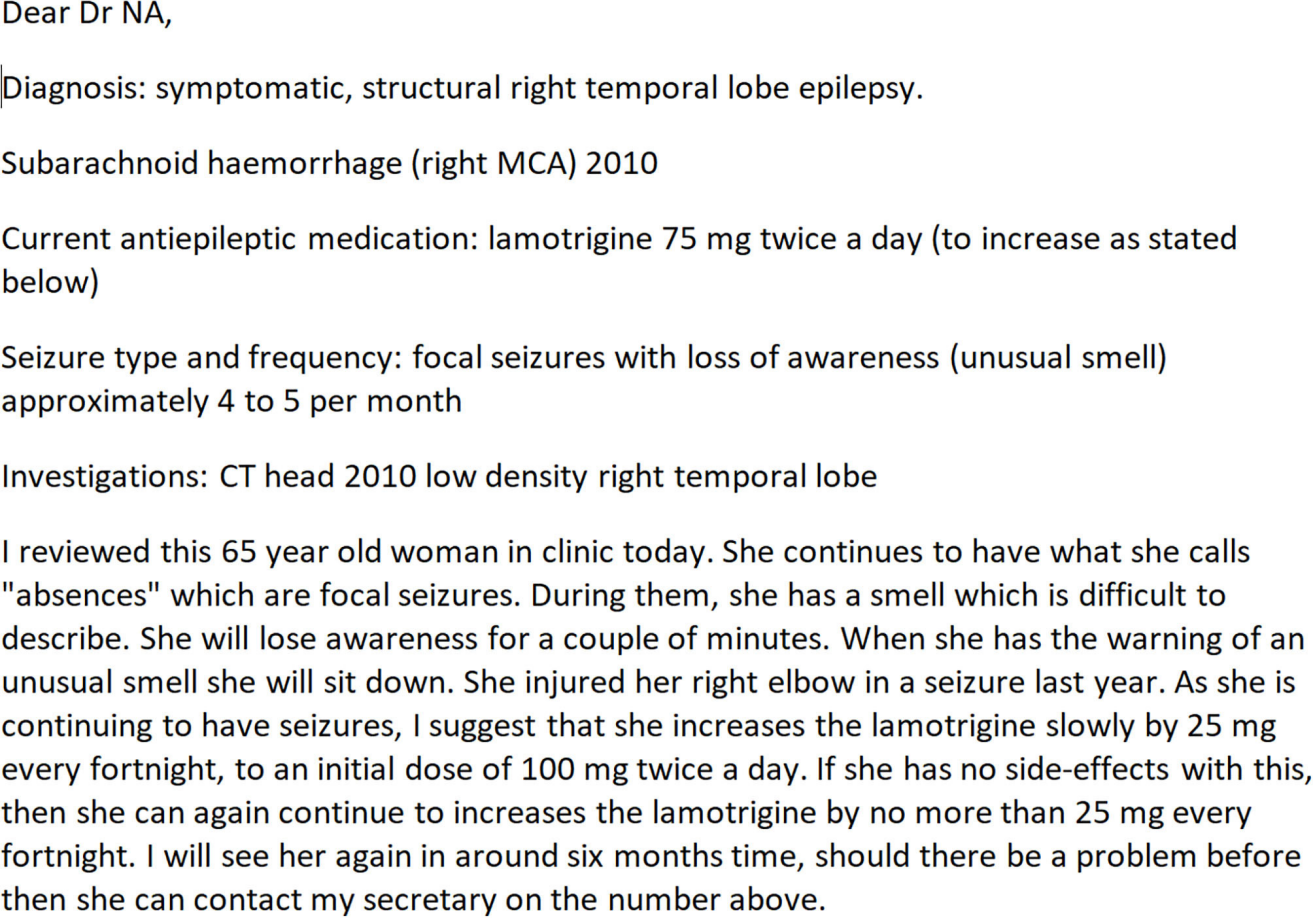
A de-identified and anonymised unstructured free-text clinical letter.

Across various domains, there are increasing quantities of unstructured free-text data being produced. For example, in healthcare, clinical letters are created to document details of patient consultations. These letters often contain valuable information relating to patient symptoms, history, risks, outcomes, prescriptions, and diagnoses, as shown in Figure 1. However, the unstructured nature of these letters poses a challenge for the retrieval and utilisation of embedded data in novel healthcare research. Whilst Natural Language Processing (NLP) techniques can be employed to perform information extraction, gold standard annotation datasets are still necessary for training and validation.

It is therefore important to provide domain experts with the tools necessary to annotate unstructured text rapidly and accurately. Support from such tools could consist of highlighting key phrases, capturing detailed attributes within a phrase, or suggesting domain-specific annotations (1).

Several tools have been developed with the aim of assisting annotators throughout the annotation processes. The brat rapid annotation tool (brat) is widely used and allows users to annotate both entities and attributes within entities (e.g., negation status), and define linkage, or relationships between entities (2). Some tools, such as the extensible Human Oracle Suite of Tools and Knowtator, include the ability to annotate against pre-existing ontologies such as UMLS. Like brat, these tools are run locally on user machines (3, 4).

More recent annotation tools, such as the brat based WebAnno, TeatTat, and Marky have introduced useful features that emphasise distributed annotation tasks (5–7). Users can set up projects with annotation schemas and are able to compute inter-annotator agreement across sessions.

Machine learning has been used in some tools to speed up the annotation process by providing annotation suggestions to the user. These approaches fall into two categories: pre-annotation and active learning. The Rapid Text Annotation Tool generates pre-annotations from a gold standard annotated corpus. INCEpTION and ezTAG use active learning, adapting to user annotations and improving suggestions over time (8, 9). There is also a growing emphasis on web-based software that does not rely on local installations, as shown by tools such as Anafora (10).

The structured annotations that result from annotating a document with an annotation tool can be used as the building blocks of datasets for training and developing NLP tools and Artificial Intelligence (AI) systems. However, it takes an experienced annotator an average of 15–30 min to annotate a document that has 41 data elements embedded (11). The time-consuming nature of this process, combined with the value of annotator time, can make it infeasible for individual groups to annotate the quantities of documents necessary to train large-scale, accurate AI systems. As such, opportunities to utilise the information embedded in unstructured documents for NLP and AI may be limited.

Markup aims to incorporate desirable features from existing tools, whilst introducing novel features to assist annotators and streamline the annotation process. The primary novel feature offering of Markup is an extension to traditional active learning based Named Entity Recognition (NER) suggestions. Markup uses Long Short Term Memory (LSTM) networks to perform sequence-to-sequence conversion of sentences into relevant attributes for named entities. The networks are trained on synthetically generated templates *via* a bespoke data generation interface offered by Markup. Markup is fully integrated with UMLS and uses phrase approximation to map annotations to UMLS Concepts. The tool is available as a central resource *via* a website, https://www.getmarkup.com/, and as a local application (no internet required) which can be installed from https://github.com/samueldobbie/markup/.

## Materials and Methods

### Display

**Figure 2:**
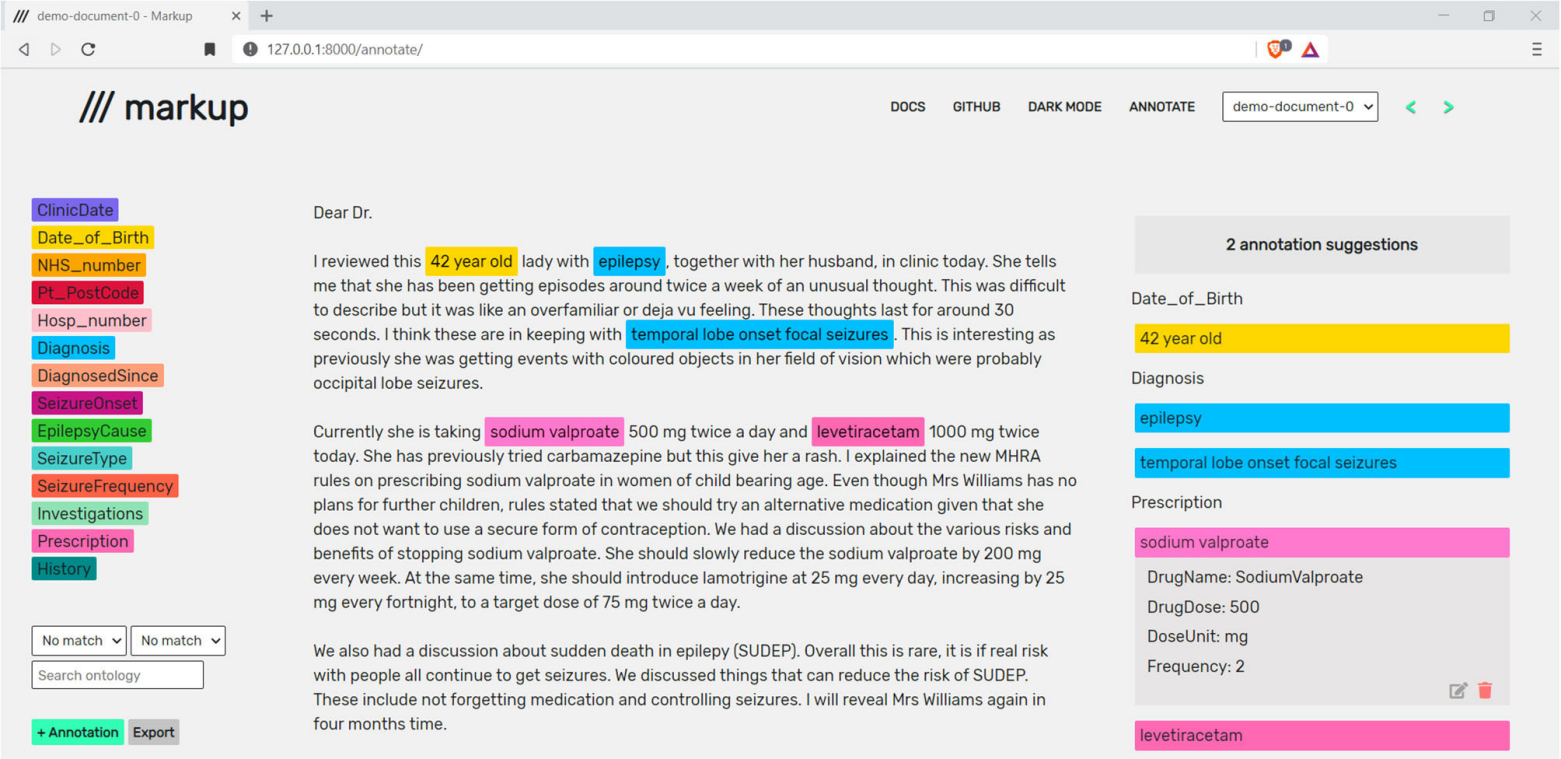
A live annotation session in Markup. Note that the document shown is a synthetic letter that has been created by a clinician to reflect real-world clinic text.

Markup provides a compartmentalised display consisting of a configuration panel (left), a document panel (centre), and an annotation panel (right), with the option of a dark mode (Figure 2).

#### Configuration Panel

The configuration panel contains selectable entities and attributes parsed from the user-defined configuration file. The panel also lists suggested ontology mappings and acts as the central hub for adding and exporting annotations.

#### Document Panel

The document panel contains the text of the currently opened document. Spans of text the user has annotated will be coloured based on the selected entity, as described in section Annotation.

#### Annotation Panel

The annotation panel displays a categorised and ordered list of annotations added by the user along with predicted annotations, as discussed in section Predictive Annotation Suggestions.

### Setup

**Figure 3:**
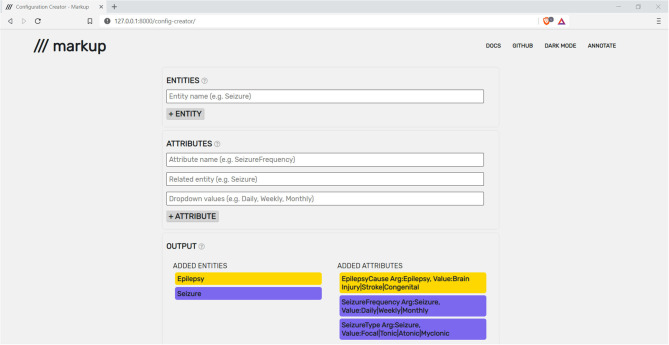
Markup's built-in configuration file creator being used to construct a file with epilepsy and seizure entities and attributes.

Markup has a quick and intuitive setup process, which enables users to open, navigate, and annotate any number of plaintext documents during a single session. To define the entities and attributes that will be available throughout the annotation task, a configuration file in brat standoff format must be provided. To assist users, Markup offers an in-built configuration file creator that outputs in brat standoff format (Figure 3).

Existing annotation files may be specified during setup, with the existing annotations being displayed for each related document. These annotation files must have the .ann file extension and be in brat standoff format.

Users have the option of uploading a custom ontology to be used for automated ontology mappings and direct querying, as discussed in section Automated Ontology Mapping. The custom ontology must be a plain text file, with each line being of the form [TERM][TAB][CODE]. If no custom ontology is provided, pre-loaded ontologies, such as UMLS, may be used. Certain pre-loaded ontologies require additional user permissions, thus users may need to authenticate *via* an external account, such as a UMLS account, prior to access. Markup allows users to authenticate via the UMLS authentication API.

### Annotation

Markup annotations can be added by selecting a span of text within a document along with the desired entity and any number of attributes. Annotation placement is unaffected by existing annotations, and thus complex data can be captured *via* repeated annotation of the same region.

Given the intricate details required for capture within documents, configuration files can be complex and lengthy, making them difficult to navigate throughout the annotation process. Markup provides an intuitive alternative by dynamically displaying attributes based on the selected entity, thus preventing irrelevant options from appearing in the users' view. Each attribute also allows for free-text input, enabling a user to capture attributes that have not been previously defined in the configuration file.

Adding an annotation will colour the annotated text based on the colour of the corresponding entity and the annotation will be added to the categorised display in the annotation panel, as shown in Figure 2. Information related to each annotation (e.g., attribute values) can be viewed by selecting the desired annotation in the annotation panel, or by hovering over an annotation with the cursor. Additional options (e.g. delete or edit an annotation) can also be seen upon selection of an annotation within the annotation panel.

Upon completion of the annotation process, the user can export the annotations into the brat standoff format, thus enabling external NLP software, such as the General Architecture for Text Engineering (12), to make use of annotations produced by Markup.

### Predictive Annotation Suggestions

Manually adding an annotation is a time-consuming task across all domains, despite annotations often following predictable formats. For example, when annotating a sentence that contains a prescription, it is likely that the sentence will contain a drug name, dose, unit, and frequency.

**Figure 4:**
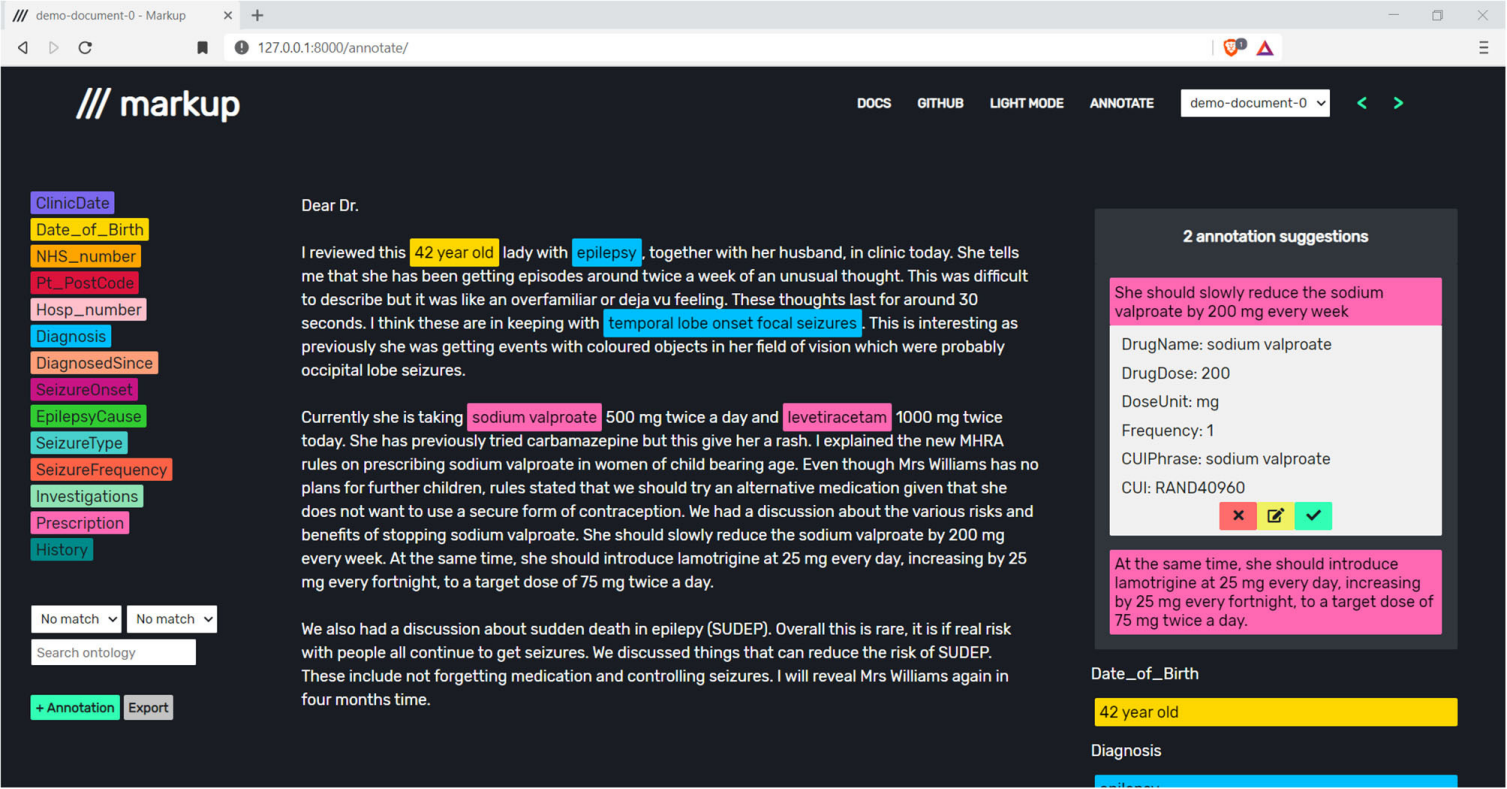
Accepting, editing, or rejecting prescription suggestions to store annotations and improve future annotation suggestions. Note that the document shown is a synthetic letter that has been created by a clinician to reflect real-world clinic text.

During the annotation process, Markup learns to automate the process of identifying, suggesting, and adding complex annotations, with appropriate entities and attributes. Markup will continuously suggest annotations for the document within the users' view (Figure 4).

**Figure 5:**
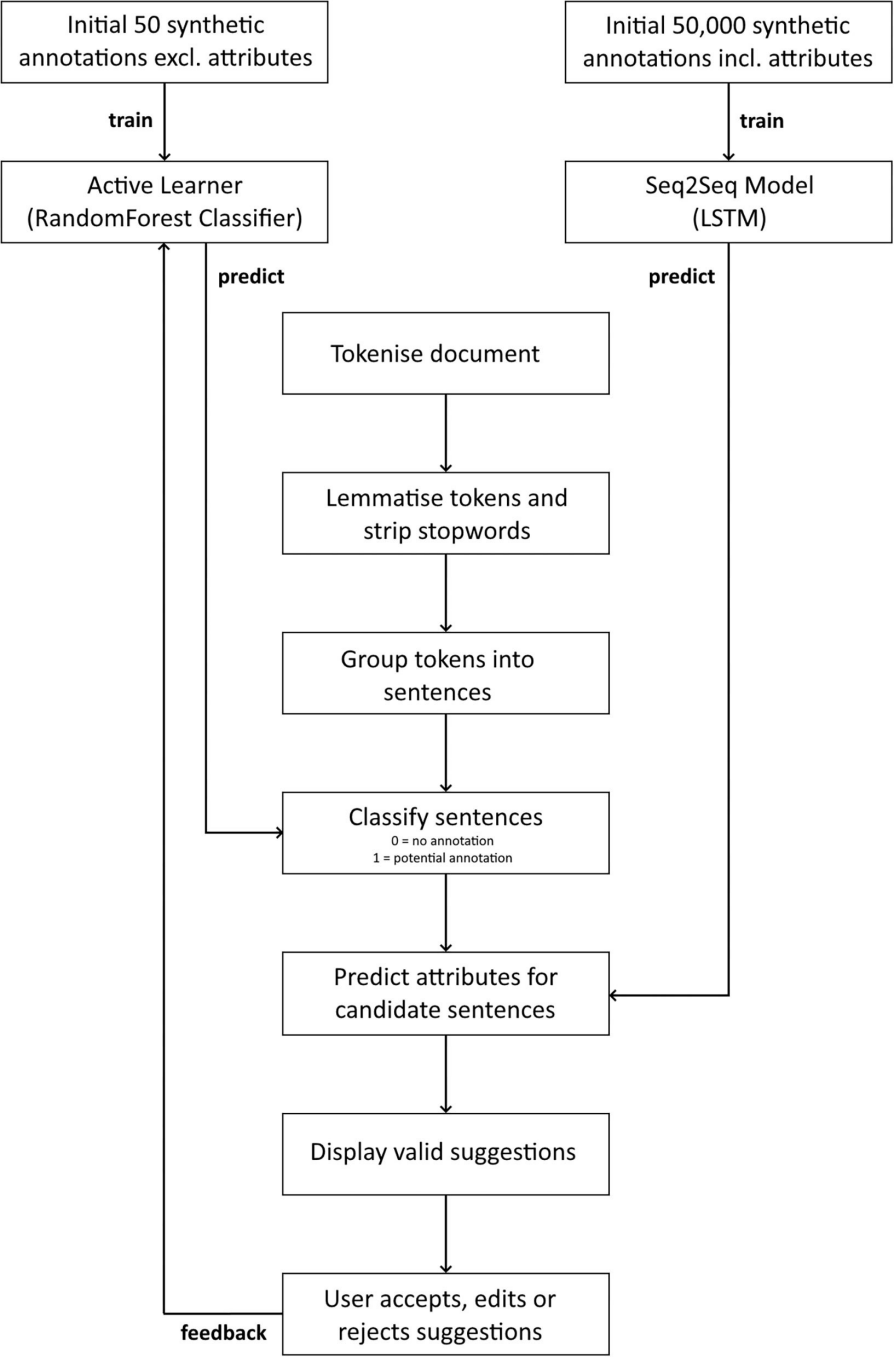
A flow diagram of the annotation suggestion process. Named Entity Recognition is performed using an Active Learner with an underlying Random Forest classifier, and sentence to attribute conversion is performed using an LSTM-based Sequence-to-Sequence model.

Markup achieves this using active learning (AL) and sequence-to-sequence (Seq2Seq) models. The AL model uses an underlying Random Forest classifier to identify target sentences, such as sentences that contain prescriptions (13). The word-level Long Short-Term Memory (LSTM) based Seq2Seq model converts each target sentence into relevant attributes (14–16). The models are continuously adjusted based on feedback from user interactions with the predicted annotation suggestions (Figure 5).

We have demonstrated prediction of prescription-based annotations. Markup is however unrestricted in its annotation predictions and offers functionality that enables users to generate their own synthetic data and train models with no technical expertise.

### Automated Ontology Mapping

Misspellings and unconventional wordings often contained within documents make it beneficial to map annotated terms to standardised and recognised versions of a similar term in external ontologies, as shown in Table 1.

**Table 1:** Mappings of terms, as found in a clinical letter, to standardised versions of the terms in an external ontology.

**Annotated term**	**Mapped ontology term**
Focal eeppilepsy	Focal epilepsy
Patient broke their arm	Broken arm
ADR	Adverse drug reaction

Markup assists annotators in the production of normalised annotation datasets by providing functionality for automatically mapping annotations to standardised terms in both existing ontologies, such as UMLS, and user-defined ontologies. Upon selecting a span of text within Markup, a dropdown list is automatically populated with all relevant matches from the chosen ontology.

To achieve this, the lexical similarity is computed between the span of text being annotated and the terms within the ontology, using Cosine Similarity. To account for misspelt words, terms are broken down into n-chars before computing the similarity. To avoid the computational expense of repeatedly converting all terms to n-chars and computing the similarity between the span of text and all terms within the ontology, SimString has been used (17). With SimString, a database is constructed containing the n-char representations of each term, and an initial filtering is performed upon inputting the selected span of text, with Cosine Similarity then being computed against the remaining terms. The terms that surpass the similarity threshold are then ordered by relevance within the dropdown list by computing the Levenshtein Distance between the terms and the selected span of text.

Whilst the above process occurs automatically each time a user selects a span of text, users also have the option of manually querying the ontology *via* Markup, which will populate the dropdown list based on the input search term. If a mapping is accepted by the user, all data relevant to that item within the ontology, such as its unique identifier, will be associated with the annotation.

### Safety and Security

Markup makes use of the Transport Layer Protocol (via HTTP over TLS) to encrypt data that is communicated between the client and Markup server(s). Given the broad range of documents that Markup may be used to annotate, it is recommended that a local installation and server is used when annotating sensitive documents, or where organisational policy dictates that data may not leave a local environment or organisation, to help minimize security risks.

### Comparing Active Learning and Manual Annotation

We used Markup's built-in data generator to synthetically generate 50 training samples (medication prescriptions) as a seed for the AL and 50,000 for the Seq2Seq model. The data generator allows users to define multiple terms of interest such as subsets of UMLS concepts, arbitrary strings and number range fields that can be used to define attribute variables within template sentences. These template sentences are then resampled, where attribute values are permuted to generate a set number of training samples. All 50,000 training samples contained target outputs for the Seq2Seq model, whereas the samples for the AL contained no target output, and were simply split into positive and negative samples, with 25 being positive samples (sentences containing prescriptions) and 25 being negative samples (sentences not containing prescriptions). No real-world training data was used to train the AL or Seq2Seq models.

Using the local version of Markup on standalone machines we compared annotation speed within Markup using manual annotation and active learning. Four experienced annotators each annotated the prescriptions (including drug name, dose strength, frequency, and further directions) found within two sets of 25 pseudonymised clinic letters.

One set was manually annotated and the other was annotated using active learning. Annotators 1 and 2 used manual annotation for set 1, and annotators 3 and 4 used active learning suggestions for set 1. The annotators swapped methods for set 2 with annotators 1 and 2 using active learning and annotators 3 and 4 using manual annotation. This method reduced potential bias from differences in annotation difficulty between the sets of letters. Manual annotation is generally quicker when documents have been previously viewed and so annotators used different methods for the different sets of letters.

During the active learning session, annotators could accept, edit, or reject suggestions. Where Markup did not suggest a valid prescription for a prescription phase the annotators were instructed to annotate it manually.

## Results

Markup was developed using React and TypeScript (front-end) and Python (back-end), and makes use of the Flask web framework. De-identified and anonymised clinic letters were used throughout Markup's development for validation of functional outputs.

A multi-disciplinary team of annotators, including data analysts and clinicians, provided extensive feedback whilst testing Markup on epilepsy and plastic surgery clinic letters. The captured annotations are being used to build and test real-world NLP applications, such as an updated and expanded version of ExECT (18). Re-annotation, following iterations of annotation definitions, was incorporated for flexibility. UMLS codes, certainty context, and multiple components from complex phrases were captured and exported in a structured format.

### Evaluation of Active Learning Annotation Suggestions

The use of active learning annotation suggestions reduced annotation time by 14.2% (11.01 min, 95% CI 0.62–0.87, *p* < 0.001) and increased the mean pairwise inter-annotator F-measure by 0.02 (95% CI 0.05–1, *p* = 1) (Table 2).

**Table 2:** Comparing manual and predictive annotation.

**Method**	**Letter set**	**Annotation time (minutes)**		**Mean time (minutes)**	**Annotated items N**	**Pairwise F-measure**
		**First annotator**	**Second annotator**			
Manual	1	41.24	51.22	46.23	85	0.96
	2	35.36	63	49.18	96	0.89
Predictive	1	32.33	40.46	36.40395	91	0.93
	2	33.00	41	37.00	96	0.88
			Mean decrease in time		14.2% (11.01 min) CI 0.62–0.87, *p* < 0.001)	
			Mean decrease in F		0.02 (95% CI 0.05–1, *p* = 1)	

*Each letter set contained 25 pseudo-anonymised letters. Each of 4 annotators manually annotated 1 set and used annotation predictions to annotate the other set (see Method section Comparing Active Learning and Manual Annotation). Pairwise F-measure is defined as the average of F-measures between multiple annotators where each annotator is sequentially selected as the gold standard annotator*.

One annotator tested whether the Active Learner (AL) improved suggestions over time by comparing baseline suggestions for all 25 letters compared to suggestions when given input by the annotator. The AL model reduced the number of incorrect suggestions by 52% (*N* = 50) when compared to baseline suggestions without human intervention (*N* = 106) and marginally increased the number of correct suggestions by 3% (*N* = 68) compared to the baseline (*N* = 66).

An initial system usability survey was run within the annotation team, resulting in an average score of 91. Surveys found at https://github.com/samueldobbie/markup-sus/.

## Discussion

Markup is a general-purpose, open-source, web-based annotation tool that offers a range of features to enhance the annotation experience, tackle limitations from existing tools, and reduce annotation time.

Markup has four primary strengths. The first is the inclusion of integrated ontology access and automated ontology mappings. The second is the inclusion of predictive annotation suggestions *via* an Active Learner that improves with user feedback, and a Sequence-to-Sequence (Seq2Seq) model which helps to automate the capture of annotations attributes. The third is that Markup's models can be trained without a pre-annotated corpus, as Markup's data generator enables users to define synthetic templates to produce training data. The fourth is Markup's availability as a web application, enabling annotation without the installation of software components, and as a standalone application *via* a local server.

Markup's active learning reduced annotation time by 14.2% and decreased pairwise inter-annotator F-measure by only 0.02. The time taken for the Seq2Seq to convert target sentences into annotation attributes is the largest delay in the suggestion process; taking a mean of 20.2 s across 5 random documents, which contain a mean of 55 sentences. Reducing this time will be a priority for ongoing development.

The reduced annotation time, with a negligible decrease in the pairwise inter-annotator F-measure, point to good quality suggestions provided to the user. The Active Learner is, however, sensitive to human input. Incorrect suggestions were reduced by 52%, with user input to the Active Learner, when compared to baseline pre-annotations.

The Neves systematic review identifies accurate pre-annotation as one of the main areas of focus to improve annotation tools (19). To our knowledge, most existing annotation tools that use pre-annotation and active learning based suggestions are limited to Named Entity Recognition (NER) tasks. Users then have the task of manually entering attributes for the suggested entity. Markup's annotation suggestion process automatically suggests attributes using a Seq2Seq model that has been trained on synthetically generated data.

The INCEpTION annotation platform uses Active Learning to predict and assign a label category to an annotation span that has been suggested to the user following NER in the active learning phase (20, 21). INCEpTION achieves this using uncertainty sampling when trained from a gold standard pre-annotated corpus. As Markup only requires synthetically generated sentence templates to begin suggesting complex annotations, it removes the need to pre-annotate a large corpus for training purposes, as users can simply define a set of base templates. ezTag provides an interactive learning mode that does not require training data. It allows the use of pre-trained taggers, or a string-matcher, to suggest NER to users. ezTag is similar to Markup in this respect. However, Markup goes beyond standard NER problems with its use of LSTM. This expands NER recognition into more challenging areas such as sequence mapping, and automated tagging of sub-annotations within the main annotation span.

Whilst Markup has been used successfully on complicated, real-world clinic letters, there are still several limitations that will need to be addressed during future development. The most notable limitation is the lack of user presence within the application, as no option to create a user account currently exists. As such, there is no option to share configurations, documents, annotations, and ontologies within an annotation group as is possible with tools such as Webanno (5).

The automated ontology mappings by Markup are currently limited in that suggestions are based solely upon the lexical similarity between terms, thus if the user were to select an acronym within the document, the full-form phrase is unlikely to be detected as a relevant mapping. To tackle this, future work will be done to suggest mappings based on semantic similarity *via* word embeddings.

Further work is required to expand the number of input and output formats Markup can work with, to streamline the process of integrating Markup into new and existing pipelines. For instance, Markup only works with plaintext files, and is generally aimed toward annotation of short documents such as clinic letters. Future support will include the use of Apache Tika to ingest word, pdf and non-standard document formats commonly found in healthcare such as DICOM image files.

## Data Availability Statement

The original contributions presented in the study are included in the article/supplementary material, further inquiries can be directed to the corresponding author/s.

## Ethics Statement

pseudonymised clinical letters routinely collected from the Swansea Epilepsy clinic were used throughout the development of Markup for testing purposes.

## Author Contributions

AL and SD conceived, designed, and developed Markup and drafted the manuscript. BF-S, HS, CJ, and WP contributed to data collection and/or data analyses. AA and ST provided resources and technical support. All authors revised the manuscript.

## Conflict of Interest

The authors declare that the research was conducted in the absence of any commercial or financial relationships that could be construed as a potential conflict of interest.

## Publisher's Note

All claims expressed in this article are solely those of the authors and do not necessarily represent those of their affiliated organizations, or those of the publisher, the editors and the reviewers. Any product that may be evaluated in this article, or claim that may be made by its manufacturer, is not guaranteed or endorsed by the publisher.
